# Micro-Injection Moulding of PEO/PCL Blend–Based Matrices for Extended Oral Delivery of Fenbendazole

**DOI:** 10.3390/pharmaceutics15030900

**Published:** 2023-03-10

**Authors:** Gilberto S. N. Bezerra, Gabriel G. De Lima, Declan M. Colbert, Elaine Halligan, Joseph Geever, Luke Geever

**Affiliations:** 1 PRISM Research Institute, Technological University of the Shannon: Midlands Midwest, N37HD68 Athlone, Ireland; 2 Programa de Pós-Graduação em Engenharia e Ciência dos Materiais—PIPE, Universidade Federal do Paraná, Curitiba 81531-980, Brazil

**Keywords:** hot-melt extrusion, micro-injection moulding, solid dispersion, fenbendazole, extended-release, animal health

## Abstract

Fenbendazole (FBZ) is a broad-spectrum anthelmintic administered orally to ruminants; nevertheless, its poor water solubility has been the main limitation to reaching satisfactory and sustained levels at the site of the target parasites. Hence, the exploitation of hot-melt extrusion (HME) and micro-injection moulding (µIM) for the manufacturing of extended-release tablets of plasticised solid dispersions of poly(ethylene oxide) (PEO)/polycaprolactone (PCL) and FBZ was investigated due to their unique suitability for semi-continuous manufacturing of pharmaceutical oral solid dosage forms. High-performance liquid chromatography (HPLC) analysis demonstrated a consistent and uniform drug content in the tablets. Thermal analysis using differential scanning calorimetry (DSC) and thermogravimetric analysis (TGA) suggested the amorphous state of the active ingredient, which was endorsed by powder X-ray diffraction spectroscopy (pXRD). Fourier transform infrared spectroscopy (FTIR) analysis did not display any new peak indicative of either a chemical interaction or degradation. Scanning electron microscopy (SEM) images showed smoother surfaces and broader pores as we increased the PCL content. Electron-dispersive X-ray spectroscopy (EDX) revealed that the drug was homogeneously distributed within the polymeric matrices. Drug release studies attested that all moulded tablets of amorphous solid dispersions improved the drug solubility, with the PEO/PCL blend–based matrices showing drug release by Korsmeyer–Peppas kinetics. Thus, HME coupled with µIM proved to be a promising approach towards a continuous automated manufacturing process for the production of oral solid dispersions of benzimidazole anthelmintics to grazing cattle.

## 1. Introduction

Livestock animals (e.g., cattle, sheep, goats, swine, and poultry) are responsible for providing one-third of human protein intake. Unfortunately, these animals are susceptible to a variety of parasites, most of which can affect the quantity and quality of their derived products [1]. It has been estimated that more than 500 million ruminants are infected with helminth parasites, which have caused economic losses of over USD 3 billion every year around the world [2]. Moreover, many of these parasitic worms are transmittable to humans, which is a major cause of human morbidity [3]. Hence, effective and safe methods for controlling these organisms will benefit both farmed animals and the wider farming community [4].

Fenbendazole is a benzimidazole methylcarbamate molecule with efficacy against a broad spectrum of parasitic worms, and selective toxicity [5,6]. According to the Biopharmaceutics Classification System, FBZ is a class II drug, which is characterised by low solubility and high permeability [7]. It has been hypothesised that this poor solubility of FBZ is caused by its planar structure, leading to strong stacking interactions in the crystal lattice, and reinforced by intermolecular hydrogen bonds [8].

Benzimidazoles are commonly administered orally to ruminants as a single dose, or as a multiple-dose regimen [9]. However, their poor water solubility has been the main limitation to reach satisfactory and sustained levels of the active ingredient at the site of the target parasite [5], aiming at triggering irreversible damage [10]. As gastrointestinal nematodes are more exposed to recycled drugs that return to the rumen and small intestine, compared to unabsorbed drugs passing down the gastrointestinal tract in food, drug particles must be dissolved in enteric fluids to facilitate their absorption through the gastrointestinal mucosa [5,11].

Hot-melt extrusion has emerged as a novel processing technology in the repertoire of pharmaceutical scientists to enhance the solubility of class II drugs [12]. During the melt processing, the active pharmaceutical ingredient (API) and polymers are transferred by rotating screws through a heated barrel to form a solid solution. Upon exiting the die, the molten mixture is continuously pumped, and rapidly solidifies [13]. As a consequence of the extrusion process, the crystalline structure of the active ingredient is converted to its amorphous state and dispersed at the molecular level in the polymeric matrix, increasing the possibility of molecular interactions [14]. When compared to its crystalline form, not only does the oral administration of an amorphous benzimidazole to cows promote a faster and complete absorption, but half a dose is also enough to produce an efficacy similar to that of the drug in its crystalline state [5,15].

A matrix tablet is the simplest and most cost-effective method for fabricating an oral dosage form, which is usually made by polymers either alone (hydrophilic matrix or hydrophobic matrix) or in conjugation (hydrophilic–hydrophobic matrix systems) [16]. Currently, the application of a single polymer has been replaced by polymer blends in the development of drug delivery systems as a simple variation of a polymer:polymer blend ratio provides a large spectrum of properties [17]. Grehan et al. [18] melt-processed blends of PEO and PCL to study the release of 4-acetamidophenol. Lyons et al. [19] applied a hot-melt processing technique to manufacture monolithic matrices of PEO and PCL to carry carvedilol. Bezerra et al. [20] using melt-extruded matrices of PEO and PCL showed that the drug release can be tailored by altering the ratio of PCL. Due to their thermoplastic properties and low melting points, PEO and PCL can be extruded and moulded using conventional thermoplastic processing techniques, attending the requirements of an extended-release drug delivery system [13].

Micro-injection moulding of thermoplastic polymers targets the manufacture of microstructure parts under heat and pressure with high precision. It is a repetitive process in which the material melts inside the plasticising chamber and is injected into a microstructure mould to be shaped. µIM is a promising melt manufacturing technique for large-scale replication of microparts, and is one of the most suitable processes for replicating microstructures with medium to large production scales [21]. Moreover, it aggregates other properties in the development of extended-release drug delivery systems, such as drug particles that are uniformly embedded in the polymeric carrier, generally with low porosity, with the ability to maintain physical integrity, and with effective release control [22].

HME and µIM are novel pharmaceutical manufacturing techniques with high potential to become either continuous or semi-continuous processes in the production of plasticised solid dispersion tablets, and able to scale up to mass production [23]. The US Food and Drug Administration highlighted the advantages of implementing continuous pharmaceutical manufacturing, such as its reduced cost, increased safety, lower processing time, improved efficiency, and consistent quality [24].

Though HME and µIM have been extensively applied in the plastic processing industry, they are relatively new techniques in the pharmaceutical industry. This study broadens the understanding of the subject and proposes a semi-continuous method for the manufacture of amorphous solid dispersions to other benzimidazole anthelmintics. This is applied using previous knowledge acquired from the processing of PEO and PCL incorporated with FBZ as extruded formulations, which are used as model compounds for the production of extended-release oral dosage forms manufactured by a two-step process using HME and µIM [20,25].

Thus, the thermal, physical, chemical, and mechanical properties of the HME + µIM extended-release tablets were assessed by DSC, TGA, FTIR, pXRD, and shore D hardness. Their morphology and drug dispersion were analysed using SEM with EDX, and drug release was evaluated using UV–Vis.

## 2. Materials and Methods

### 2.1. Materials

Fenbendazole (methyl *N*-(6-phenylsulfanyl-1*H*-benzimidazol-2-yl)carbamate) was obtained from Molekula (Darlington, UK), poly(ethylene oxide) was purchased from Alroko^®^ (Hamburg, Germany), and polycaprolactone (CAPA^®^ 6506) was supplied by Perstorp (Warrington, UK). All other chemicals used in this study were of analytical grade. The chemical structures of FBZ, PEO, and PCL are shown in Table 1.

### 2.2. Hot-Melt Extrusion

Melt compounding was carried out on a benchtop Prism™ TSE 16 twin-screw co-rotating extruder, with 16 mm diameter and 15:1 length to diameter (*L*/*D*)-ratio screws (Thermo Electron Corporation, Staffordshire, UK), a speed of 50 rotations per minute, and a torque of 20–25%. An annular (rod-shaped) die was attached to the end of the barrel. Prior to compounding, individual components were weighed, placed in a sealed polyethylene bag, manually tumble-blended for 5 min, and dried in an oven for 24 h at 40 °C to minimise any degradation as a result of absorbed moisture. The samples were fed into the extruder using an automatic feeder at a rate of 9 g min^−1^ with the barrel temperature at 110 °C for the physical mixture of PEO 95% and FBZ 5% (*w*/*w*) [25], and 70 °C for neat PCL. The extrudates were air-cooled, and they were granulated using a Prism™ TSE systems granulator (Thermo Electron Corporation, Staffordshire, UK). The composition of the solid dispersion formulations (SDFs) to be moulded is presented in Table 2.

### 2.3. Melt Flow Index

The melt viscosity behaviour of extruded granules was evaluated using a Rosand Melt Flow Indexer (Labquip Ireland Ltd., Dublin, Ireland) with a fixed weight of 2.16 kg. Prior to processing, all samples were dried in an oven for 24 h at 40 °C. The granules were tested at 80, 100, and 120 °C with the material flowing through an orifice of 2.0 mm in diameter for 10 min, 6 samples were collected, and results were reported in g 10 min^−1^.

### 2.4. Micro-Injection Moulding

**Figure 1:**
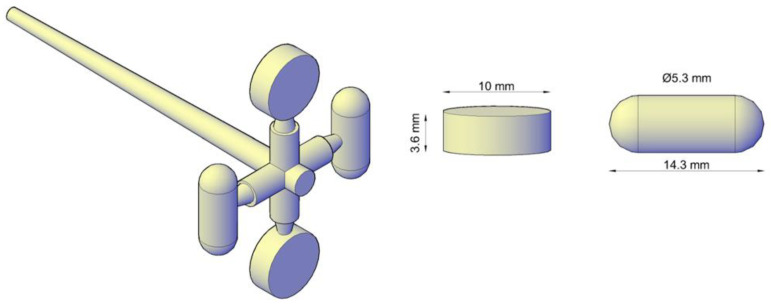
Dimensions of the µIM cavities in the shape of tablet and capsule modelled in Autodesk AutoCAD 2021.

Moulded tablets were manufactured using the Babyplast 6/10P µIM machine (Cronoplast, S.L., Barcelona, Spain). It possesses three heating zones (plasticising, chamber, and nozzle) with the plasticising chamber separated from the piston by the presence of thirteen ball bearings leading to a non-aggressive melting by heat conduction [29]. The processing conditions set for the manufacturing of the tablets can be summarised as (i) temperature-controlled areas—100 (plasticising), 100 (chamber), and 90 °C (nozzle); (ii) shot size based on the volume of material necessary per shot to fill sprue, runners, gates, and cavities—16 mm; (iii) cooling time required for the molten material to solidify—45 s; (iv) 1st and 2nd injection pressure—70 and 60 bar, respectively; (v) 1st and 2nd injection pressure time—2 and 6 s, respectively; (vi) decompression—4 mm. Prior to processing, extruded granules were sieved to obtain similar particle sizes, and dried in an oven for 24 h at 40 °C. The µIM tool consists of four cavities, two cylindrical capsules, and two round tablets, presented in Figure 1. Nevertheless, this work was limited to the tablet dosage form with a final concentration of FBZ in SDF 1 of 5%, SDF 2 of 3.25%, and SDF 3 of 2.75%.

### 2.5. Shore D Hardness Evaluation

A digital shore scale D durometer hardness tester was used to perform the hardness evaluation from the moulded tablets (CV Instruments Ltd., England, UK), which was based on the penetration of a sharp conical point from the shore apparatus on the sample surface, under a load of 4 kg. The values reported in this study are the mean of ten tablets from each formulation.

### 2.6. Thermal Characterisation by DSC and TGA/DTG

DSC analysis was carried out using a Pyris 6 DSC (PerkinElmer, Waltham, MA, USA), which was calibrated using indium as the reference material. Samples between 6 and 8 mg were accurately measured and placed in lid-sealed aluminium pans. Calorimetry scans were performed using a heating rate of 10 °C min^−1^ applying standard heat from 30 to 280 °C min^−1^ for the API and heat/cool/heat cycles of 30/100/30/280 °C min^−1^ for neat polymers and moulded tablets. Samples were tested under nitrogen atmosphere with a flow of 30 mL min^−1^ to avoid oxidation.

TGA analysis was performed using a Pyris 1 TGA (PerkinElmer, Waltham, MA, USA). Thermogravimetric curves were obtained using 10 mg of the sample in aluminium pans, using a heating rate of 10 °C min^−1^ from 30 to 700 °C min^−1^, under nitrogen atmosphere with flow of 20 mL min^−1^.

Both DSC and TGA/DTG measurements were conducted using Pyris–Instrument Managing Software (PerkinElmer, Waltham, MA, USA).

### 2.7. Attenuated Total Reflectance-Fourier Transform Infrared Spectroscopy

The samples spectra were performed on a PerkinElmer Spectrum One with a universal ATR sampling accessory (Waltham, MA, USA) at room temperature, in the spectral range from 650 to 4000 cm^−1^, with 4 scans per sample and a compression force of 85 N.

### 2.8. Powder X-ray Diffraction Spectroscopy

Powder diffraction data (pXRD) were collected on a Bruker D2 Phaser (Bruker AXS, Karlsruhe, Germany) with Cu Kα radiation (λ = 1.54178 Å) at 30 kV and 10 mA, using a 1.0 mm primary slit, 3 mm air scatter gap, 2.5° Soller module, and Ni filter for Kβ radiation, using a LynxEye detector. Samples were loaded on a silicon single-crystal zero-background sample holder with a cavity of 24.6 mm × 1 mm. Data were collected in the range from 5 to 55° in 2θ, using a 0.01° step size and 0.5 s per step.

### 2.9. Scanning Electron Microscopy with Energy-Dispersive X-ray Spectroscopy

Injection-moulded tablets were evaluated using a scanning electron microscope (Tescan Mira, Oxford Instruments, Cambridge, UK) for the examination of their external surface topography, and internal area after cracking, followed by energy-dispersive X-ray spectroscopy (X-Max, Oxford Instruments, Cambridge, UK) for the assessment of their chemical composition and drug disposition uniformity. Prior to imaging, samples were sputtered with gold utilising a Baltec SCD 005 sputter coater (BAL-TEC GmbH, Schalksmühle, Germany).

### 2.10. Determination of Drug Content

The content uniformity studies were performed on three moulded tablets. Each tablet was crushed and placed in a 50 mL volumetric flask, and dimethyl sulfoxide was added and shaken for 10 min. Further dimethyl sulfoxide was added to the solution to make up the volume. An aliquot (1 mL) of this solution was diluted to 1 mL of mobile phase, filtered using PTFE 0.45 μm, and analysed using HPLC.

Chromatographic analyses were carried out using a system consisting of a Waters Alliance e2695 separations module combined with a Waters 2487 dual λ absorbance detector (Waters Chromatography Ireland Ltd., Dublin, Ireland) according to [30]. A Thermo Scientific^®^BDS Hypersil C8 column (250 mm × 4.60 mm, 5 µm) (Fisher Scientific Ireland Ltd., Dublin, Ireland) maintained at ambient temperature was used as the stationary phase. The mobile phase consisted of methanol and 0.025 M monopotassium phosphate (70:30 *v*/*v*) adjusted to pH 3.20 using ortho-phosphoric acid, filtered, and degassed. A flow rate of 1 mL min^−1^ was maintained during the procedure, the detector was set at 288 nm, and the sample injection volume was 10 µL.

A stock standard solution was prepared by dissolving 20 mg of FBZ in 100 mL of methanol with a concentration of 200 µg mL^−1^, which was diluted to plot a calibration curve of area versus concentration with R2 of 0.9995, and used to determine the drug concentration in each moulded tablet.

### 2.11. Release Studies

In vitro drug dissolution studies of the moulded tablets were carried out using a Distek dissolution apparatus 1 (baskets) (North Brunswick, NJ, USA). Each formulation was tested in triplicate in a dissolution vessel containing 500 mL of dissolution medium. The literature states that benzimidazole anthelmintics are weakly basic drugs showing a pH-dependent solubility behaviour [31], which means they should be less water-soluble at ruminal pH (5.5–6.5) than at abomasum pH (2–3) [5,32]. Hence, the dissolution tests were performed in acetate buffer (pH 5.5) and hydrochloric acid (pH 2), at 39 ± 0.5 °C, and a stirring speed of 100 rotations per minute to address the pH-sensitivity of FBZ as well as to evaluate the stability of moulded tablets at these simulated ruminal and abomasum conditions [33,34]. The buffer solutions were prepared according to the U.S. Pharmacopeia USP 32 [35].

At predetermined intervals, samples of 2 mL were withdrawn, filtered using PTFE 0.45 μm, replaced by fresh media, and then measured using the UV–Vis spectrophotometer 1280 (Shimadzu, Kyoto, Japan) using a quartz cuvette at 288 nm. A stock standard solution was prepared by dissolving 5 mg of FBZ in 100 mL of methanol with a concentration of 50 µg mL^−1^, which was diluted to plot a calibration curve of absorbance versus concentration with R2 of 0.9994. The amount of drug dissolved was determined using the calibration curve.

DDSolver is a free-of-charge software program, available as an add-in program for Microsoft Excel [36]. It was used to select a suitable model-dependent approach for fitting our dissolution data based on the adjusted coefficient of determination (*R*^2^_adjusted_), the Akaike Information Criterion (AIC), and the Model Selection Criterion (MSC), followed by the application of two model-independent approaches: (i) the difference factor ($f_{1})$—a measure of the relative error between two curves (Equation (1)); (ii) the similarity factor ($f_{2})$—a measure of the similarity in the percent of dissolution between two curves (Equation (2)).

(1) $$f_{1}=\left[\frac{\sum_{t=1}^{n}\left|R_{t}-T_{t}\right|}{\sum_{t=1}^{n}R_{t}}\right]\times 100$$

(2) $$f_{2}=50\cdot \log\left\{\left.{\left[1+\frac{1}{n}\sum_{t=1}^{n}{\left(R_{t}-T_{t}\right)}^{2}\right]}^{-0.5}\times 100\right\}\right.$$

$R_{t}$ and $T_{t}$ correspond to the percentage dissolved of the reference and test profile, respectively, at time point t, and n is the number of sampling points. Both equations are endorsed by the FDA [37]. For the profiles to be considered similar, ($f_{1})$ should be less than 15 (0–15) and ($f_{2})$ greater than 50 (50–100) [36,37].

### 2.12. Percentage Mass Loss of Moulded Tablets

After dissolution studies, the tablets were dried in an oven for 24 h at 40 °C, and their percent of mass loss was calculated considering *W*_1_ as the weight before dissolution and *W*_2_ as the weight after dissolution testing, according to the following equation (Equation (3)):

(3) $$Mass\;Loss\;\left(\%\right)=\frac{W_{1}-W_{2}}{W_{1}}\times 100$$

### 2.13. Statistical Analyses

Statistical analyses were performed using the Minitab^®^ 20.3 statistical software program for Windows. A one-way analysis of variance (ANOVA) was used to compare the mean values of weight, hardness, and uniformity of drug content among the moulded tablets, followed by the Tukey method for pairwise comparisons. The means were considered significantly different at *p* ≤ 0.05, with a confidence level of 95%.

## 3. Results

### 3.1. Material Melt Viscosity by MFI

MFI tests were carried out on extruded granules that composed SDF 1, SDF 2, and SDF 3 at 80, 100, and 120 °C to study both the thermal and mechanical effect of the melt extrusion process on their viscosity properties, and to define the optimum temperature range to µIM tablet-shaped parts.

The MFI result correlates the increase in the flow with the reduction in the melt viscosity from certain polymers; therefore, a higher MFI is required in order to allow the molten material to flow through small-size sprue, runners, and gates at high speed and pressure [38].

**Figure 2:**
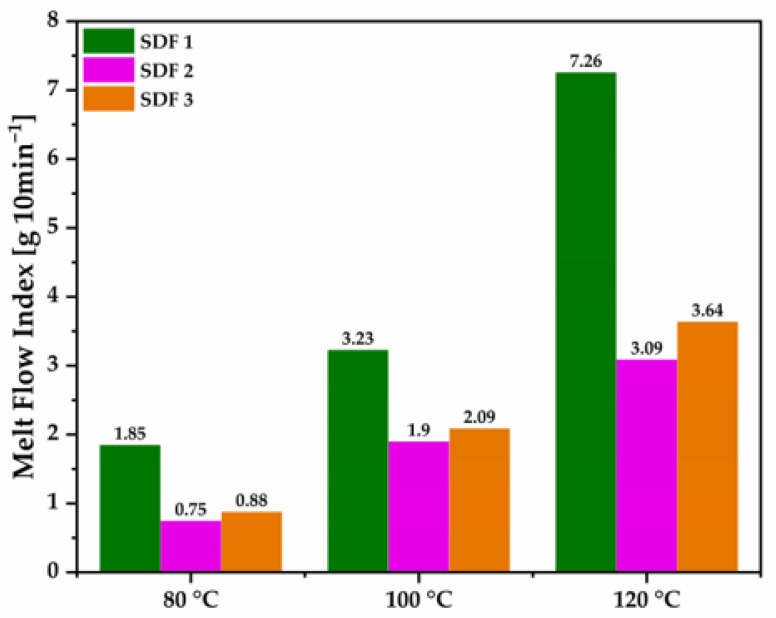
Melt flow index readings from extrudate granules of SDF 1, SDF 2, and SDF 3.

In Figure 2, SDF 1’s melt viscosity increased from 1.85 g 10 min^−1^ to 7.26 g 10 min^−1^ as the temperature increased from 80 to 120 °C. In a previous work, neat PEO-extruded granules demonstrated much lower melt viscosity values, increasing from 0.48 g 10 min^−1^ to 2.40 g 10 min^−1^, as the temperature increased from 80 to 120 °C [20]. In other words, FBZ acts as a plasticiser when melt extruded with PEO, probably due to a good miscibility, decreasing the polymer melt viscosity and improving the molten material flow, a requirement for the moulding process [14,19,39].

Meanwhile, Grehan et al. [18] revealed that the incorporation of a polymer such as PCL, that is known for its low melt viscosity, enables polymers such as PEO recognised by its high melt viscosity to be melt-extruded easily, which was later endorsed by Bezerra et al. [20]. Nevertheless, comparing the MFI results from SDF 2 and SDF 3 to SDF 1, it is clearly seen that the addition of PCL granules increased the melt viscosity properties of the molten material, likely because they were not melt-extruded together, which did not allow PCL to act as a plasticiser for PEO. As they are immiscible polymers, only a subtle improvement in the melt viscosity properties was achieved when increasing the amount of PCL from 35 to 45%. It should also be highlighted that when two polymers such as PEO and PCL have different viscosity properties, the less viscous polymer usually tends to encapsulate the more viscous one, resulting in a blend richer in the most viscous component [40], which is expected for our moulded tablets as extruded granules of PEO 95% + FBZ 5% (*w*/*w*) demonstrated in this study a MFI of 3.23 g 10 min^−1^ at 100 °C, while extruded granules of neat PCL showed in a previous one a MFI of 4.31 g 10 min^−1^ at 100 °C [20].

Thus, the µIM machine manufacturer recommends a 10 to 15% higher temperature at the plasticising zone, followed by lower temperatures at the chamber and nozzle zones [29]. Based on our MFI data, we could define the conditions for melt-processing thermal stable solid dispersion tablets, which is described above in the methodology section.

### 3.2. Micro-Injection Moulding Tablets

**Figure 3:**
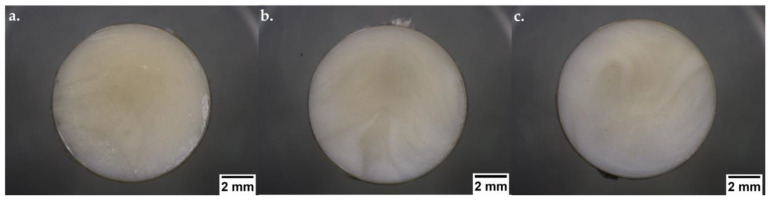
Digital photographs (ShuttlePix Digital Microscope, Nikon Corporation, Tokyo, Japan) displaying the physical aspects of µIM tablets: (**a**) SDF 1, (**b**) SDF 2, and (**c**) SDF 3.

A µIM machine was used for the production of plasticised solid dispersion tablets. After manufacturing, independent of their composition, the moulded tablets displayed a similar appearance with a smooth surface, opaque colour, and extreme hardness due to their semi-crystalline nature and thermoplastic properties from PEO and PCL. The physical characteristics of the moulded tablets are presented in Figure 3.

The weight of the moulded tablets was evaluated to confirm the consistency of the process over time. It should be stressed that the weight control has been considered an easy and economical strategy to identify variances during mould processing [38].

Table 3 displays the results of the weight variation of ten tablets with standard deviations lower than 1% of the overall tablet weight, which is in accordance with a pharmacopeial requirement for the limit of variation of ±5% [41]. There was a clear correlation between the reduction in the weight of the tablets, followed by the addition of PCL, which was confirmed by the one-way ANOVA resulting in *p* < 0.05 and endorsed by Tukey’s test.

Shore D hardness measurements from moulded tablets revealed an association between the incorporation of PCL inside the polymeric matrices and higher hardness values from 49.85 (SDF 1) to 50.43 (SDF 2) and 52.23 (SDF 3) Shore D, being considered statistically distinguishable with *p* < 0.05, and attested by Tukey’s test after pairwise comparisons that SDF 1’s hardness was significantly different from those of SDF 2 and SDF 3. In other words, though the addition of PCL reduced the weight of the moulded tablets, it led to higher hardness values, which could favour the tablet’s physical stability inside the cow’s reticulo-rumen system.

The uniformity content of FBZ in the moulded tablets was performed using HPLC. All solid dispersion formulations showed a single peak in the chromatograms with a retention time of approximately 6.80 min, which means that the moulding process did not affect the API properties, which could have led to its degradation. The FBZ content in the moulded tablets ranged from 90.47 to 100.09% with a standard deviation lower than 6%, demonstrating a consistent and uniform drug content, and no significant differences (*p* > 0.05). Additionally, it reveals the efficiency of this two-step manufacturing process using HME and µIM. SDF 3 was the only formulation to meet the requirements of the USP guidelines for FBZ dosage forms, where a tolerance of the drug content should be not less than 98% and not more than 101% [42]. Nevertheless, by optimising the injection moulding parameters individually for each formulation, it should provide more drug content within the moulded tablets.

### 3.3. Thermal Study of the Moulded Tablets by DSC and TGA

**Figure 4:**
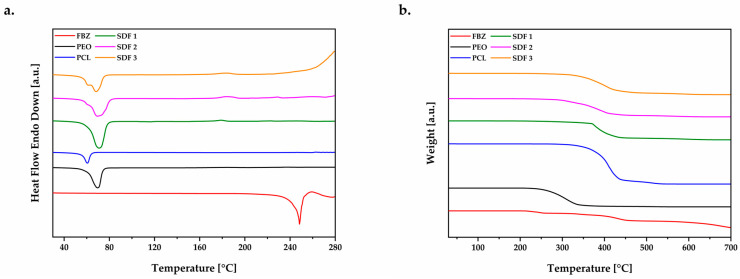
(**a**) DSC and (**b**) TGA thermograms of FBZ, PEO powder, PCL powder, SDF 1, SDF 2, and SDF 3.

Calorimetric (Figure 4a) and thermogravimetric (Figure 4b) analyses were carried out to investigate the physical state of the solid dispersion moulded tablets after the melt manufacturing techniques.

The thermogram pattern of FBZ was typical of a highly crystalline material, characterised by a sharp endothermic peak at 245 °C in *T*_onset_ 235 °C (Δ*H****_m_*** = 115 J g^−1^) corresponding to the drug melting transition, followed by decomposition. Additionally, FBZ was demonstrated to be stable up to 164 °C, when the first stage of decomposition is marked by the loss of the sulphur atom [43], and has to be taken into consideration before exposing the drug to higher melting processing temperatures.

DSC analyses of the moulded solid dispersions 1, 2, and 3 revealed a single melting peak corresponding to the crystalline fraction of PEO at temperatures of 67 °C in *T*_onset_ 58 °C (Δ*H****_m_*** = 137 J g^−1^), 67 °C in *T*_onset_ 57 °C (Δ*H****_m_*** = 107 J g^−1^), and 65 °C in *T*_onset_ 56 °C (Δ*H****_m_*** = 72 J g^−1^), respectively. As the fraction of PCL increased from 35% to 45% inside the formulations, it was possible to identify a small “shoulder” besides the PEO melting peak due to their immiscibility [26,44]. The decrease in the heat of fusion amount was already indicative of the crystallinity change within the system. Whilst TGA analysis showed that moulded tablets of SDF 1 were able to increase the drug thermal stability from 164 °C to 187 °C, moulded tablets composed of PEO/PCL blends (SDF 2 and SDF 3) increased from 164 °C to 183 and 190 °C, respectively.

The absence of the drug phase transition can be attributed to the capacity of PEO/PCL blends during the melt-processing to promote a depression on the API’s chemical potential leading to a melting process below its usual temperature. Therefore, the drug crystalline structure was likely converted into its amorphous state, establishing new intermolecular interactions, which reveals the good miscibility of the drug with the polymeric carrier [20]. Hence, these formulations are likely composed by amorphous FBZ dispersed among the amorphous polymer chains of the semi-crystalline PEO [45,46] encapsulated by amorphous/semi-crystalline PCL [40], which agrees with the MFI results that PCL is not acting as a plasticiser, but as another encapsulating agent for FBZ.

These results are similar to the thermal analysis of the extruded solid dispersions approached in our previous works [20,25], which ratifies that the thermal (heat) and mechanical (shear) treatments during the extrusion and µIM processes did not affect the solid state of the systems.

### 3.4. Physical and Chemical Evaluation of the Moulded Tablets by FTIR and pXRD

FTIR analysis was performed to investigate intermolecular interactions between FBZ and the polymeric carrier after exposure to thermal and mechanical stresses during the melt processing techniques.

**Figure 5:**
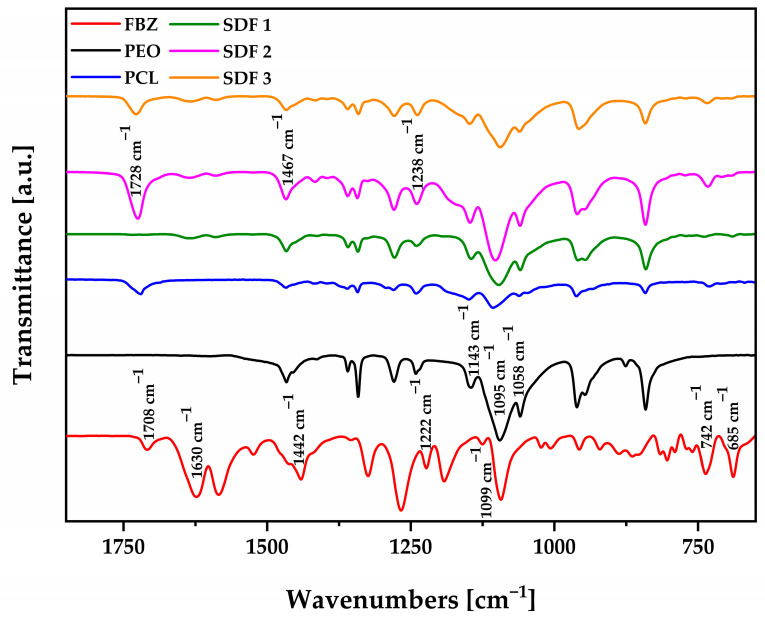
FTIR spectra of FBZ, PEO powder, PCL powder, SDF 1, SDF 2, and SDF 3.

Figure 5 shows six IR spectra corresponding to FBZ, PEO powder, PCL powder, SDF 1, SDF 2, and SDF 3. The FBZ spectrum was first confirmed with the SpectraBase™/Wiley (CAS #43210-67-9), followed by its comparison to the spectra of the moulded formulations.

Despite FBZ’s high hydrophobicity, it is capable of accepting and donating hydrogen bonds [27], where it is expected to intensify the level of intermolecular interactions with the polymeric carrier, particularly PEO due to the thermomechanical mixing effects from the extruder. After comparing the FBZ spectrum to the spectra of SDF 1, SDF 2, and SDF 3, the presence of new peaks was not evidenced, which would suggest either a chemical interaction between the API and PEO/PCL or their degradation due to the melt manufacturing processes.

Nevertheless, an expected reduction in the API bands intensity was noticed, due to their small content within the polymeric matrices, at 1630 cm^−1^, 1099 cm^−1^, 742 cm^−1^, and 685 cm^−1^, followed by dislocation of the signal from 1442 cm^−1^ to 1467 cm^−1^, 1708 to 1728 cm^−1^, and 1222 to 1238 cm^−1^. In another study, the authors reported that subtle differences in the drug’s main peaks can be related to its crystallinity reduction as it was converted to the amorphous state [47], indicating the drug–polymer mixing at the molecular level [48].

PEO is a hydrophilic polymer with the presence of a -C-O-C absorption complex at 1143, 1095, and 1058 cm^−1^, which represents the combination of ether and methylene groups [47], being very sensitive to polymer chain conformation changes, and capable of hydrogen intermolecular interactions with FBZ. Hence, the notorious changes in the shape, intensity, and shift of these absorption bands are clearly consequences of drug and polymer interactions [47]. Moreover, PEO hydrophobic properties have been well documented [49], as the majority of its vibration peaks are attributed to methylene groups at 1467 cm^−1^, 1359 cm^−1^, 1342 cm^−1^, and 1276 cm^−1^ [47,49], increasing the possibility of hydrophobic interactions with FBZ.

PCL is a versatile hydrophobic polymer with some polarity in its structure from groups that vibrate at 1728 cm^−1^ (C=O), 1238 cm^−1^ (COO), as well as 1099 and 1047 cm^−1^ (C-O), allowing the establishment of intermolecular hydrogen bonds with FBZ [20]. Moreover, the presence of several methylene groups at 1465, 1407, and 1362 cm^−1^ contributes to the formation of hydrophobic interactions between them [50].

These findings support our calorimetric data, indicating the good miscibility between FBZ and the polymeric carrier, mainly PEO, through the establishment of intermolecular interactions between them, which is essential for the successful conversion of the drug to its amorphous form.

To further investigate the amorphous nature of the API inside the polymer carriers and their interactions, pXRD was employed in this study through qualitative analyses to obtain more information required to support our results.

**Figure 6:**
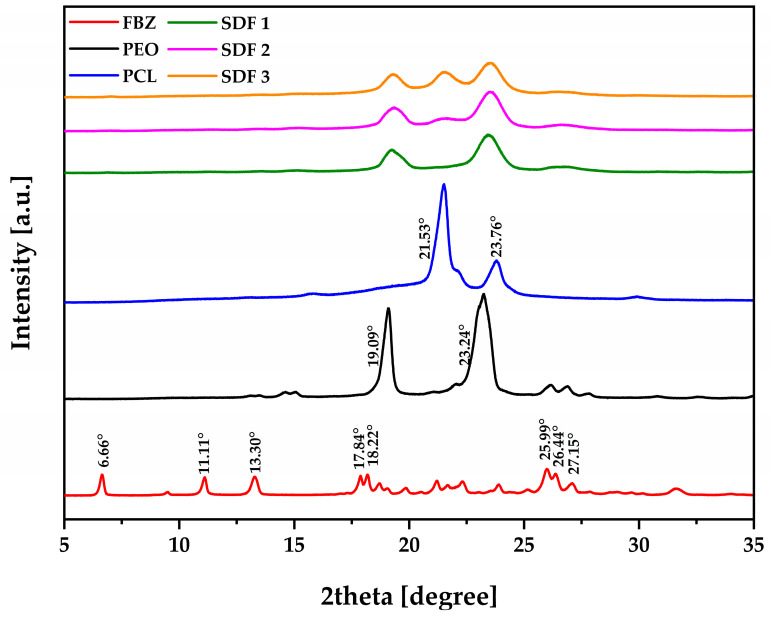
XRD diffraction patterns of FBZ, PEO powder, PCL powder, SDF 1, SDF 2, and SDF 3.

The results obtained from the FBZ diffractogram (Figure 6) revealed its crystalline nature with eight well-evidenced peaks appearing at 2θ = 6.66, 11.11, 13.30, 17.84, 18.22, 25.99, 26.44, and 27.15° with similar results found in the literature [20,51]. Despite not seeing any additional crystalline peak in the X-ray diffraction patterns obtained from the moulded formulations, the main ones related to the drug crystalline nature were completely missing, confirming its amorphous state. After analysing the diffractogram of each polymer before and after the melt processing techniques, PEO’s main diffraction peaks at 19.09 and 23.24° [52] reduced expressively, followed by PCL diffraction peaks at 21.53 and 23.76° [53] almost disappearing, but the diffraction peak at 21.53° improved slightly due to the increase in PCL inside the formulations from 35 to 45%. The literature reports that PEO/PCL-blend–based matrices usually display convoluted peaks with intensities dependent on the blend composition [26]. Thus, these data endorse our thermal studies.

### 3.5. Drug Dispersion Assessment by SEM and EDX

**Figure 7:**
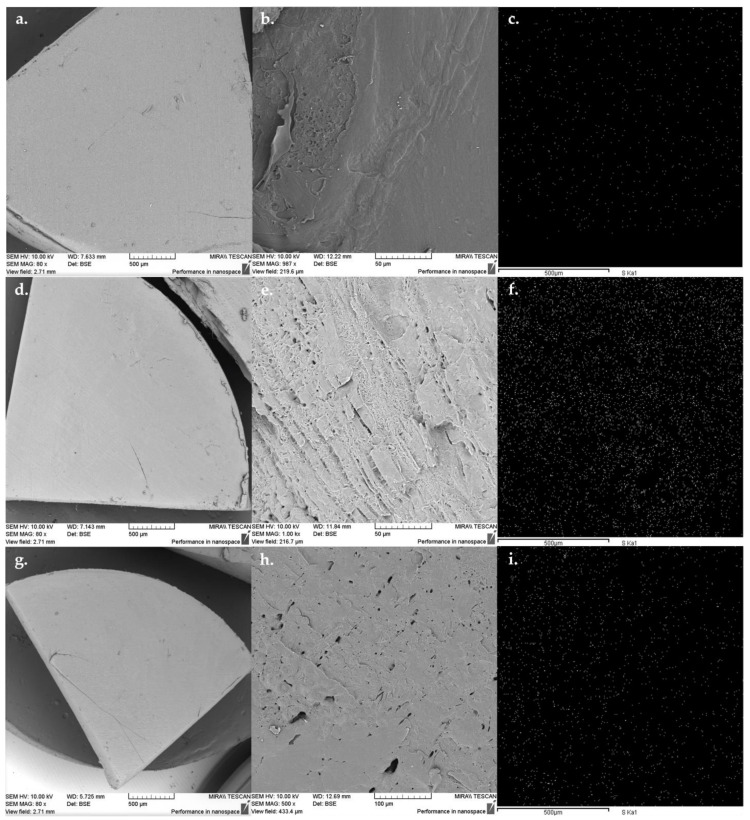
SDF 1 (**a**,**b**), SDF 2 (**d**,**e**), and SDF 3 (**g**,**h**) images of the external and internal surfaces from moulded tablets, followed by SDF 1 (**c**), SDF 2 (**f**), and SDF 3 (**i**) spectra revealing the dispersion of FBZ identified by the element sulphur throughout the polymeric matrices.

Moulded tablets were analysed using SEM to explore their topography and morphology. SDF 1 (Figure 7a,b), SDF 2 (Figure 7d,e), and SDF 3 (Figure 7g,h) displayed a homogeneous and smooth external surfaces as a result of the good coalescence of the molten material inside the mould [54], but they displayed a few fissures that could likely be prevented through further optimisation of some process parameters, such as injection pressure, holding pressure, and cooling time [45,55].

On the one hand, the fractured surface morphology from SDF 2 displayed a very rough surface with pores of different sizes and shapes (Figure 7e), and as we increased the proportion of PCL from 35 to 45%, the surface became smoother and the pores became broader (Figure 7h). Such pores are usually connected by ducts leading to the formation of a dense network [44,56]. On the other hand, the SDF 1 image revealed a slightly rough internal surface without visual access to the presence of pores (Figure 7b), which is typical of moulded tablets from a single polymer matrix to present poor porosity [57].

The FBZ dispersion throughout the polymeric matrices was assessed by EDX for SDF 1 (Figure 7c), SDF 2 (Figure 7f), and SDF 3 (Figure 7i). FBZ has an atom of sulphur in its molecular structure [27], while PEO and PCL are represented by atoms of carbon, oxygen, and hydrogen. After EDX spectra analyses of the moulded solid dispersion formulations, only the sulphur atom present in the chemical structure of the drug was mapped, with its uniform dispersion and immersion inside the polymeric carrier being distinctly perceptible.

Thermal processing of polymeric matrices and active ingredients can decrease the free volume, reduce the pore radius, increase the degree of packing, and create a more complex pore network, which can lead to a reduction in the drug release rates [54,56]. As our formulations had been subjected to additional shear and temperature due to the two-step process using HME and µIM, it is expected to have caused some positive effect on the ability of the polymeric matrices to reduce the drug release rates. Thus, in vitro dissolution studies are required.

### 3.6. Release Studies

The dissolution profiles from the moulded solid dispersion tablets were evaluated at pH 2 and pH 5.5 to address the pH-sensitivity of FBZ as well as to investigate their stability at these simulated ruminal and abomasum conditions.

**Figure 8:**
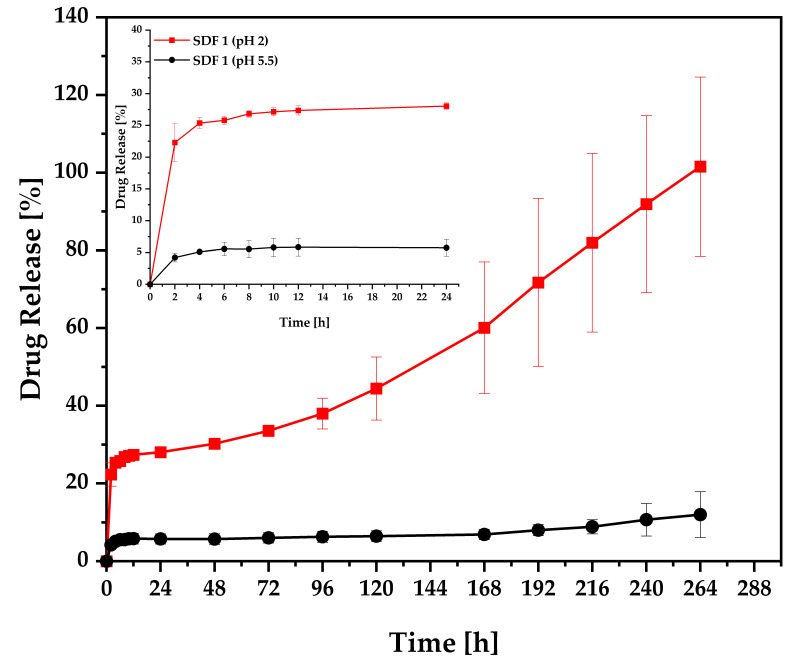
Dissolution profiles of moulded tablets of SDF 1—PEO 95% + FBZ 5% (*w*/*w*) evaluated under pH 2 and pH 5.5.

The dissolution test of moulded tablets from SDF 1 was carried out under pH 2, revealing a fast drug release of 22% in 2 h, followed by a slower drug release achieving 28% in 24 h. When submitted to a different condition at pH 5.5, the tablets displayed a fast drug release of only 4% in 2 h, followed by a subtle increase to 5% in 24 h (Figure 8). Surov et al. [58] evaluated the dissolution properties of the free base of FBZ in acidic pH, reporting that the solubility level was not enough to be quantified within 6 h of the experiment. Therefore, a slight difference in the drug solubility can have a major effect on its bioavailability. This represents that, independent of pH, the HME + µIM tablet composed of PEO was successful in improving FBZ’s poor dissolution properties.

The main release mechanism of PEO is classified as anomalous, in which the delivery of the API is a consequence of polymer swelling, drug diffusion, and matrix erosion, showing faster drug release rates when higher polymer concentrations are included [56]. In a previous study, the extruded formulation of PEO and FBZ exhibited complete drug release in 3 h [25]. Moulded tablets are expected to have a slower drug release [38], followed by a lower mass loss rate, when compared to extruded formulations due to their different geometries and density characteristics that contribute to poor solvent penetration [57]. Hence, it was expected that moulded tablets of PEO and FBZ would show extended drug release and longer breakdown rates. Moreover, due to FBZ’s basic nature (pKa 5.12), it was expected that the imidazole ring at acidic pH would ionise and improve substantially the drug solubility [59]. Therefore, the test was carried out at a prolonged time period for a better comprehension of FBZ dissolution properties, achieving a complete drug dissolution under the acidic environment in 11 days.

In this case, the monolithic matrix of PEO was responsible for physically stabilising the amorphous drug, forming a solid solution between them, and to sustain the supersaturation achieved during the dissolution assay for a certain period of time [12]. However, PEO is a highly hydrophilic polymer, and it usually takes a few hours to achieve complete breakdown of the dosage form [25], losing its capacity to sustain the supersaturation of the amorphous drug in the medium. As the polymeric carrier dissolves very fast, so does the drug, leading to an initial drug supersaturation, followed by nucleation, recrystallisation, and precipitation [12,60]. Therefore, we believe that the drug release profile was initially controlled by the polymeric matrix, but later by the dissolution kinetics of the crystallised drug [60], showing total redissolution of the precipitate, probably because of the small size of the crystals as well as the fact that they were optimally wetted as they originated from the supersaturated solution [61]. This precipitation phenomenon related to a benzimidazole molecule with polyethylene glycol in an acidic environment has been reported previously by Mukherjee et al. [62]. Thus, SDF 1 was not tested for the drug release kinetic analysis and comparison of dissolution profiles.

**Figure 9:**
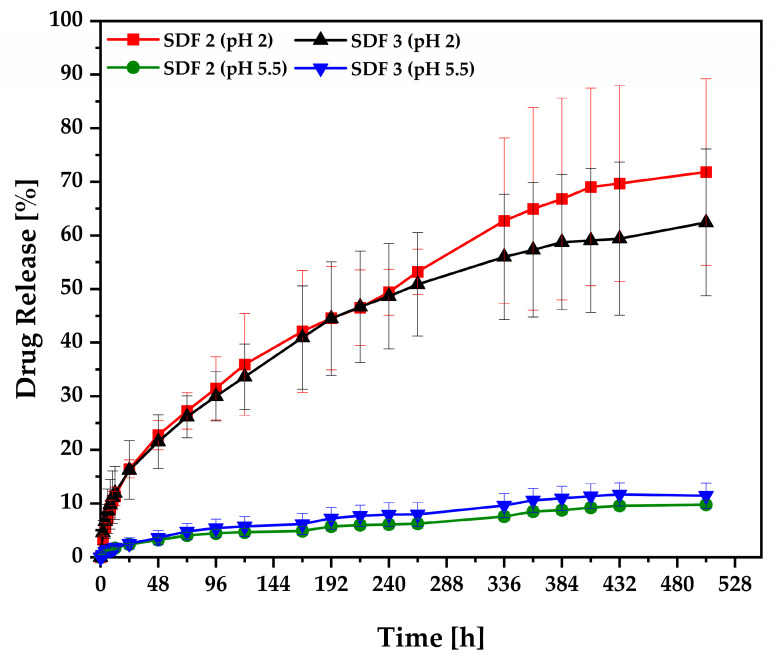
Dissolution profiles of moulded tablets of SDF 2—PEO/FBZ 65% + PCL 35% (*w*/*w*), followed by SDF 3—PEO/FBZ 55% + PCL 45% (*w*/*w*) evaluated under pH 2 and pH 5.5.

In Figure 9, the dissolution profile of the moulded SDF 2 carried out under pH 2 is displayed, achieving 3% of drug release in 2 h, 16% in 24 h, 42% in 7 days, and 71% in 21 days. At a different pH (5.5) of the dissolution medium, this solid dispersion had a drug release of 2% in 24 h, 4% in 7 days, and 9% in 21 days, whilst the percentage of drug dissolved from moulded SDF 3 performed under pH 2 was 4% in 2 h, 16% in 24 h, 40% in 7 days, and 62% in 21 days. The same formulation evaluated at pH 5.5 released 2% in 24 h, 6% in 7 days, and 11% in 21 days. Under the same conditions, neat FBZ dissolution properties were analysed, achieving 2% of drug dissolved at pH 2 and 0% of drug dissolved at pH 5.5 after 24 h of the experiment. In other words, it is clearly evident that FBZ is a pH-dependent drug capable of ionisation in an acidic environment, which contributes to its dissolution along with the drug amorphization. However, FBZ absorption will depend on two factors: (i) the drug lipid solubility to favour its passive membrane absorption through the mucous surface, and (ii) the drug degree of ionisation as it needs to be in the unionised state to be absorbed [5]. After being released in the abomasum, we believe that FBZ molecules could be extensively absorbed through the small and large intestines, where the pH ranges between 6 and 8 [32], influencing the drug to its unionised form [63]. Thus, parasitic worms would be affected by the unabsorbed drug passing down the gastrointestinal tract and, mainly, by prolonged exposition to the recycled drug secreted back in the abomasum and small intestine [5].

Particularly, HME + µIM solid dispersions composed of PEO/PCL blend–based matrices were demonstrated to be an efficient strategy to improve FBZ solubility (SDF, 2~35 times; SDF, 3~31 times) as well as to extend its release rates for 3 weeks.

Drug release kinetics from FBZ-moulded tablets were evaluated by various mathematical models (Table 4). The dissolution data were fitted to zero-order, first-order, Higuchi, and Korsmeyer–Peppas to determine the mechanism of drug release. The FBZ release (in pH 2) from SDF 2 and SDF 3 showed a good fit into the Korsmeyer–Peppas equation, presenting the *R*^2^_adjusted_ of 0.99 and 0.99, AIC of 85.60 and 87.49, and MSC of 5.68 and 5.29, respectively, based on the following criteria: *R*^2^_adjusted_ (the highest), AIC (the lowest), and MSC (the highest) [36]. This model is often used to describe the drug release behaviour from polymeric systems, combining the effect of diffusion and erosion mechanisms for drug release. Thus, the values of the diffusional exponent (*n*) for both formulations were 0.51 and 0.45, respectively, which was interpreted as a non-Fickian release or anomalous transport of drug (0.45 < *n* < 0.89), coupling diffusion/polymer relaxation [64,65]. Based on this model, we can assume that the FBZ release mechanism is mediated by drug diffusion/swelling (upon hydration) along with gradual erosion of the matrix, whilst the FBZ release (in pH 5.5) from SDF 2 and SDF 3 showed a good fit into Higuchi equation based on the *R*^2^_adjusted_ of 0.99 and 0.99, AIC of 87.34 and 102.20, and MSC of 5.61 and 4.65, respectively. According to this model, FBZ’s release mechanism is largely governed by diffusion through pores in the matrix filled with water [66], which is in agreement with the SEM morphology of the tablets.

A comparison of dissolution profiles was performed using a model-independent approach based on dissimilarity (*f*_1_) and similarity (*f*_2_) factors. For the dissolution profiles to be considered “similar”, the *f*_1_ value should be closer to 0 but not more than 15, and the *f*_2_ value should be closer to 100 but not less than 50 [37].

The value of *f*_1_ was 8 for the comparison of the dissolution profiles from SDF 2 and SDF 3 performed at pH 2, whereas the value of *f*_1_ was 22 for the comparison of their dissolution profiles performed at pH 5.5. Analysing the similarity factor for comparison of the dissolution profiles from SDF 2 and SDF 3 performed at pH 2, the value of *f*_2_ was 66, while for the samples performed at pH 5.5, the value of *f*_2_ was 88. Based on this information, we can assume that the dissolution profiles from SDF 2 and SDF 3, under pH 2, can be considered “similar”, presenting an *f*_1_ value of 8 and *f*_2_ value of 66, which matches with the profiles seen from the drug release.

### 3.7. Weight Loss of Moulded Tablets

The moulded tablets from SDF 2 and SDF 3 were recovered after 21 days of dissolution testing in hydrochloric acid (pH 2) and acetate buffer (pH 5.5), at 39 ± 0.5 °C and a stirring speed of 100 rotations per minute, to evaluate their physical stability at these simulated abomasum and ruminal conditions, respectively.

**Figure 10:**
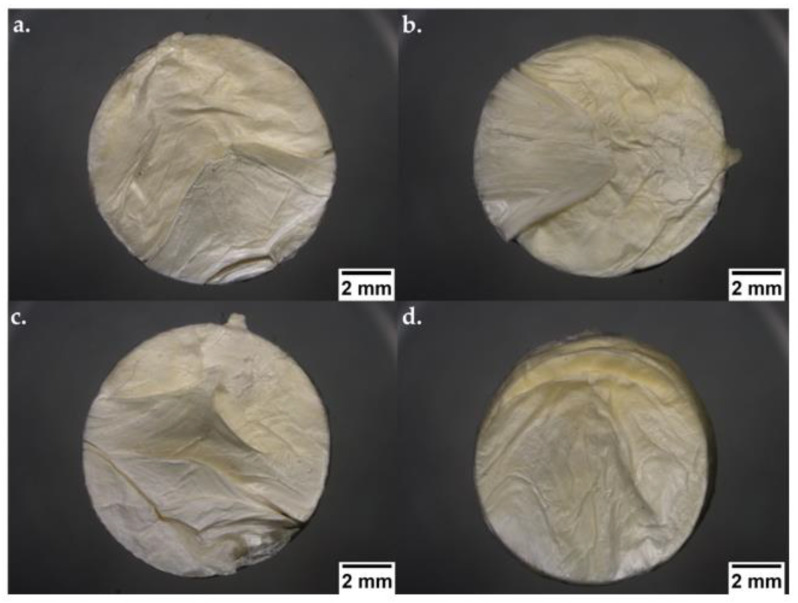
Digital photographs (ShuttlePix Digital Microscope, Nikon Corporation, Tokyo, Japan) of moulded tablets based on PEO and PCL blends after exposure to the dissolution medium at pH 2: (**a**) SDF 2 and (**c**) SDF 3, and pH 5.5: (**b**) SDF 2 and (**d**) SDF 3.

In Figure 10, digital photographs at pH 2 of SDF 2 (a) and SDF 3 (c) and at pH 5.5 of SDF 2 (b) and SDF 3 (d) are seen. In general, all formulations retained their physical shape, showing a non-homogenous colour distribution, numerous cracks, detachment of macroscopic fragments, and surface roughness.

**Figure 11:**
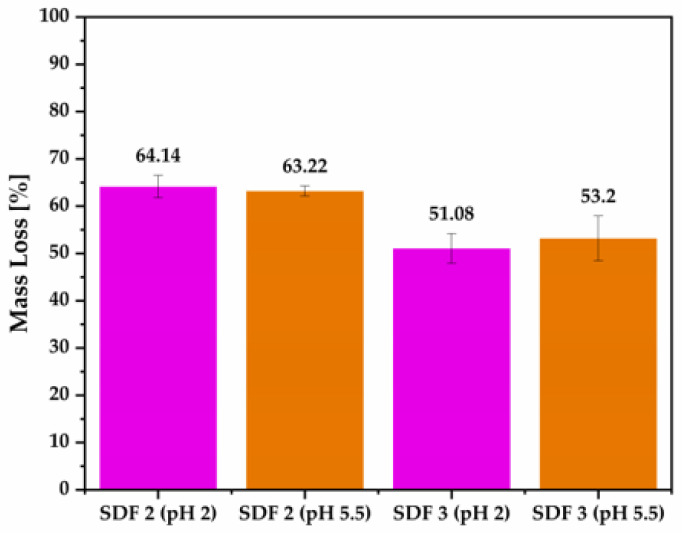
Percentage of mass loss from moulded tablets based on PEO and PCL blends after 21 days of exposition to dissolution medium at pH 2 and pH 5.5.

It is evident in the graph of mass loss (Figure 11) that higher concentrations of PCL decreased the blends susceptibility to degradation, which was expected due to its higher proportion of PCL, leading to higher crystallinity, reducing the accessibility of ester linkages and, consequently, decreasing the rate of hydrolysis [18]. This information can be endorsed by our Shore D Hardness test and pXRD data.

The percentage of weight loss of approximately 64% from SDF 2 and 53% from SDF 3, after 21 days of dissolution study, clearly indicates the complete depletion of PEO and FBZ from the moulded tablets, independent of pH, with only the structure of PCL remaining, which strengthens our hypothesis that PCL due to its lower melt viscosity was able to encapsulate PEO/FBZ. In a study conducted by Allaf et al. [26], they used water to extract PEO from compressed PEO/PCL blends, leaving only the porous structure of PCL, which reinforces our data. The Shore D hardness was also evaluated, revealing that the hardness values reduced from 50.43 to 19.75 (±SD = 1.75) Shore D for SDF 2, and 52.23 to 20.42 (±SD = 0.80) Shore D for SDF 3, which means a reduction of 39.16 and 39.09%, respectively.

Given the promising outcomes that these moulded solid dispersion formulations have shown in terms of their biopharmaceutical relevance, followed by their commercial potential in terms of scaling up using melt processing techniques, these findings can be valuable for the further development of commercial extended-release oral tablets to treat helminth infections in ruminant livestock.

## 4. Conclusions

The exploitation of HME and µIM for the manufacturing of extended-release tablets of plasticised solid dispersions of PEO/PCL and FBZ was investigated in this study, due to their unique suitability for semi-continuous manufacturing of pharmaceutical oral solid dosage forms not only for humans but also for animals.

The incorporation of PCL increased the shore D hardness values of the moulded tablets. HPLC analysis demonstrated a consistent and uniform drug content within the moulded tablets. Thermal analysis did not reveal the drug phase transition, which has been attributed to the amorphous FBZ dispersed among the amorphous polymer chains of the semi-crystalline PEO. FTIR analysis did not reveal any new peak that could indicate either a chemical interaction or degradation. X-ray analysis did not display the main diffraction peaks associated with the drug crystallinity, confirming its amorphous nature, and endorsing our thermal studies. SEM images showed that by increasing the PCL content inside the formulations, the tablet surface became smoother and the pores became broader. EDX spectra revealed a uniform drug distribution throughout the polymeric matrices. The drug release study of SDF 1 revealed drug precipitation in the acidic medium, followed by its complete redissolution in 11 days, whilst the SDF 2 and SDF 3 released 71 and 62%, respectively, in the acidic environment after 21 days of experiment, demonstrating the success of these moulded tablets in improving FBZ’s poor water solubility (SDF, 2~35 times; SDF, 3~31 times). The FBZ release (in pH 2) from SDF 2 and SDF 3 showed a good fit into the Korsmeyer–Peppas equation, and their dissolution profiles were considered “similar” according to the dissimilarity and similarity factors. The percentage of weight loss of approximately 64% from SDF 2 and 53% from SDF 3 indicates the complete depletion of PEO and FBZ from the moulded tablets, leaving only the structure of PCL, which resisted the rumen and abomasum simulated conditions.

Thus, these results provide a solid scientific foundation for the development of more robust moulded solid dispersion formulations of benzimidazole anthelmintics for grazing cattle, employing an automated, stable, and more continuous manufacturing process.

## Figures and Tables

**Table 1:** Physicochemical properties of the API and polymers [25,26,27,28].

Compound	Chemical Structure	Molecular Weight	Melting Temperature	Properties
FBZ	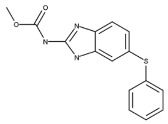	299.35 g mol^−1^	244 °C	Hydrophobic Crystalline pK_a_ (5.12, 12.72)
PEO	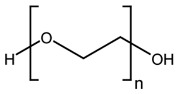	100,000–200,000 g mol^−1^	65 °C	Hydrophilic Semicrystalline Biodegradable
PCL	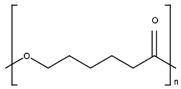	50,000 g mol^−1^	58–60 °C	Hydrophobic Semicrystalline Biodegradable

**Table 2:** Description of the composition in percentage (*w*/*w*) of the solid dispersion formulations.

Granules	SDF 1	SDF 2	SDF 3
PEO + FBZ	100%	65%	55%
PCL	0%	35%	45%

**Table 3:** Evaluation of weight, shore D hardness, and drug uniformity content of tablets fabricated using HME + µIM.

Formulation	Weight (mg)	Shore D Hardness	FBZ (%)
SDF 1	352.84 (±SD = 0.82)	49.85 (±SD = 1.68)	90.47 (±SD = 3.50)
SDF 2	347.78 (±SD = 0.37)	50.43 (±SD = 1.50)	90.94 (±SD = 2.79)
SDF3	345.25 (±SD = 2.71)	52.23 (±SD = 2.05)	100.09 (±SD = 5.25)

**Table 4:** Kinetic model selection criteria and models’ parameters.

Release Model	Equation	Parameter	SDF 2		SDF 3	
			pH 2	pH 5.5	pH 2	pH 5.5
Zero-order	$F=k_{0}\cdot t$	*R* ^2^_adjusted_	0.84	0.83	0.74	0.84
		AIC	177.55	85.00	182.39	93.71
		MSC	1.68	1.58	1.17	1.67
First-order	$F=100\cdot \left(1-e^{-k_{1}\cdot t}\right)$	*R* ^2^_adjusted_	0.95	0.84	0.89	0.86
		AIC	148.98	83.21	161.65	91.08
		MSC	2.93	1.66	2.07	1.79
Higuchi	$F=k_{H}\cdot t^{0.5}$	*R* ^2^_adjusted_	0.99	0.98	0.99	0.99
		AIC	87.34	28.18	102.20	26.77
		MSC	5.61	4.05	4.65	4.58
Korsmeyer–Peppas	$F=k_{\mathrm{KP}}\cdot t^{n}$	*R* ^2^_adjusted_	0.99	0.98	0.99	0.99
		AIC	85.60	30.15	87.49	27.46
		MSC	5.68	3.97	5.29	4.55
		*n*	0.51	0.50	0.45	0.51

*F* represents the fraction of drug released in time *t*, *k*_0_ is the zero-order release constant, *k*_1_ is the first-order release constant, *k*_H_ is the Higuchi release constant, *k*_KP_ is the release constant incorporating structural and geometric characteristics of the dosage form, *n* is the diffusional exponent indicating the mechanism of drug release [36].

## Data Availability

Not applicable.
